# Growth characteristics of freeze-tolerant baker’s yeast *Saccharomyces cerevisiae* AFY in aerobic batch culture

**DOI:** 10.1186/s40064-016-2151-3

**Published:** 2016-04-23

**Authors:** Meng Ji, Yelian Miao, Jie Yu Chen, Yebing You, Feilong Liu, Lin Xu

**Affiliations:** College of Food Science and Light Industrial Technology, Nanjing Tech University, Jiangsu, 211800 China; Faculty of Bioresource Science, Akita Prefectural University, Akita, 010-0195 Japan; College of Biotechnology and Pharmaceutical Engineering, Nanjing Tech University, Jiangsu, 211800 China

**Keywords:** Baker’s yeast, Freeze tolerance, Cell growth, Glucose, Ethanol, Kinetics

## Abstract

*Saccharomyces cerevisiae* AFY is a novel baker’s yeast strain with strong freeze-tolerance, and can be used for frozen-dough processing. The present study armed to clarify the growth characteristics of the yeast AFY. Aerobic batch culture experiments of yeast AFY were carried out using media with various initial glucose concentrations, and the culture process was analyzed kinetically. The growth of the yeast AFY exhibited a diauxic pattern with the first growth stage consuming glucose and the second growth stage consuming ethanol. The cell yield decreased with increasing initial glucose concentration in the first growth stage, and also decreased with increasing initial ethanol concentration in the second growth stage. In the initial glucose concentration range of 5.0–40.0 g/L, the simultaneous equations of Monod equation, Luedeking–Piret equation and pseudo-Luedeking–Piret equation could be used to describe the concentrations of cell, ethanol and glucose in either of the two exponential growth phases. At the initial glucose concentrations of 5.0, 10.0 and 40.0 g/L, the first exponential growth phase had a maximal specific cell growth rate of 0.52, 0.98 and 0.99 h^−1^, while the second exponential growth phase had a maximal specific cell growth rate of 0.11, 0.06 and 0.07 h^−1^, respectively. It was indicated that the efficiency of the yeast production could be improved by reducing the ethanol production in the first growth stage.

## Background

In the glucose metabolism of yeasts, glucose can be metabolized via two different energy producing pathways, i.e. oxidation or fermentation (Serio et al. 2001). The oxidative metabolism leads to cell growth, while the fermentative metabolism leads to ethanol formation. In yeast culture, therefore, the cell growth or the ethanol formation is mainly determined by the physiological state of yeast, which can be inferred from a suitable metabolic flux balance approach (Barrera-Martínez et al. 2011). In addition, the cell growth or the ethanol formation is also affected by the concentrations of glucose and oxygen in culture media. A high glucose concentration or a low oxygen concentration in culture media may result in the Crabtree effect which inhibits the cell growth and increases the ethanol formation (Win et al. 1996; Serio et al. 2001).

*Saccharomyces cerevisiae* AFY is a novel baker’s yeast strain (CCTCC NO: M2013079) with strong freeze-tolerance, recently developed by means of low temperature plasma mutation and domestication using *S. cerevisiae* CGMCC 2.1423 as the parent (Miao et al. 2014). After the cell suspension with physiological saline is stored at −20 °C for 35 days, the yeast AFY has a cell survival rate of 87.3 %, while the parent yeast has a cell survival rate of only 46.4 %. The strong freeze-tolerance of yeast AFY is attributed to its high content of intracellular-compounds such as trehalose, amino acids and glycerol in cells (Shi et al. 2014). The yeast AFY can be used for frozen-dough processing. It has been reported that, after a dough with yeast AFY is stored at −20 °C for 70 days, 60.2 % of the average fermentation rate and 87.9 % of the fermentation capacity have remained (Chen et al. 2016).

Each yeast strain has its unique cell functions, growth characteristics and nutritional requirements, based on its genome-scale metabolic network and genetic expression of transport proteins (Sainz et al. 2003; Bertilsson et al. 2008; Li et al. 2011; Lisha and Sarkar 2014). In the Erlenmeyer-flask culture, for example, the optimum culture medium for the culture of yeast AFY contains: 220 g/L glucose, 49 g/L yeast-extract, 20 ml/L inorganic-ion solution (with 60 g/L potassium phosphate monobasic, 20 g/L magnesium sulphate, 0.2 g/L calcium chloride) and 20 ml/L vitamin solution (with 2 g/L inositol, 0.5 g/L thiamine) (Chen 2015). In comparison, ordinary yeasts grow well in culture media with a sugar concentration below 138 g/L (Wang et al. 2007, 2010; Aguilar-Uscanga et al. 2011). It is considered that the yeast AFY may withstand a higher osmotic pressure than ordinary yeasts do (Zhang et al. 2015).

For the effective production of yeast AFY, it is necessary to understand its growth characteristics. In the present study, the aerobic batch culture of yeast AFY was carried out using culture media with various initial glucose concentrations. The cell growth pattern was investigated, and the simultaneous equations consisting of Monod equation, Luedeking–Piret equation and pseudo-Luedeking–Piret equation were established to describe kinetically the cell growth, the ethanol formation and the substrate consumption during the culture.

## Methods

### Inoculum preparation

The freeze-tolerant baker’s yeast strain *S. cerevisiae* AFY was preserved at 4 °C on a YPD slant culture medium (10 g/L yeast extract, 20 g/L peptone, 20 g/L glucose and 10 g/L agar) in the Food Engineering Laboratory, College of Food Science and Light Industrial Technology, Nanjing Tech University (NTU).

To prepare the inoculum, the strain was precultured in a 250 mL Erlenmeyer flask with 100 mL of the YPD culture medium (10 g/L yeast extract, 20 g/L peptone and 20 g/L glucose). The flask was kept on a rotary shaker at 30 °C and 180 rpm for 21 h. After doing this, the cell growth entered into the late exponential phase, and the inoculum contained 1.2 × 10^7^ viable cells per liter.

### Aerobic batch culture

The aerobic batch culture of yeast AFY was carried out in a 3 L stirred tank bioreactor (BIOTECH-3BG, BXBIO Co., Ltd., China) with a working volume of 1.2 L. The compositions of culture media are listed in Table 1. In the culture media, the glucose concentration was set at 1.0, 5.0, 10.0, 25.0 and 40.0 g/L respectively, and the proportions of glucose, yeast extract, inorganic-ion solution and vitamin solution to glucose were the same as those of the optimum culture medium (Chen 2015). The culture media were sterilized for 30 min at 121 °C. The conditions for the aerobic batch culture were: inoculum concentration at 5% (v/v), temperature at 30 °C, pH at 5.0 (adjusted with 2 mol/L H_2_SO_4_ and 1 mol/L NaOH), air flow at 200 L/h, dissolved oxygen (DO) level at 20 % of saturation value. The DO was controlled by the automatic variation of stirrer speed in the range of 200–800 rpm.

**Table 1:** Compositions of culture media for the aerobic batch culture

Test no.	Glucose (g/L)	Yeast extract (g/L)	Inorganic-ion solution^a^ (mL/L)	Vitamin solution^b^ (mL/L)
1	1.0	0.2	0.1	0.1
2	5.0	1.1	0.5	0.5
3	10.0	2.2	0.9	0.9
4	25.0	5.6	2.3	2.3
5	40.0	8.9	3.62	3.6

^a^The inorganic-ion solution contained 60 g/L potassium phosphate monobasic, 20 g/L magnesium sulphate and 0.2 g/L calcium chloride

^b^The vitamin solution contained 2 g/L inositol and 0.5 g/L thiamine

### Analytical methods

During the aerobic batch culture, the concentrations of cell, glucose and ethanol in the culture broth were measured in a time interval of 2 h.

The cell concentration was defined as the mass of dry yeast cells per liter of the culture broth, and measured with an optical density method (Zhang et al. 2015). Ten milliliters of the culture broth were taken, and its optical density (OD) at 600 nm was determined using a UV–Vis spectrophotometer (752S, Shanghai Lengguang Technology Co., Ltd, China). Before the measurement, the culture broth sample was diluted with deionized water if necessary. The cell concentration was calculated with the calibration equation:

1 $$C_{C} = 0.278 \times A\quad(R^{2} = 0.9923)$$

For the measurement of glucose and ethanol concentrations, 10 mL of the culture broth were taken, and centrifuged for 10 min at 3000 rpm and 4 °C. The glucose and the ethanol contained in the supernatant were analyzed using a bio-sensing system (SBA-40E, Biology Institute, Shandong Academy of Sciences, China).

For all the samples, the measurements of cell, glucose and ethanol concentrations were repeated three times, and the averages of the three measuring results were calculated.

## Results and discussion

### Cell growth pattern in aerobic batch culture

**Fig. 1:**
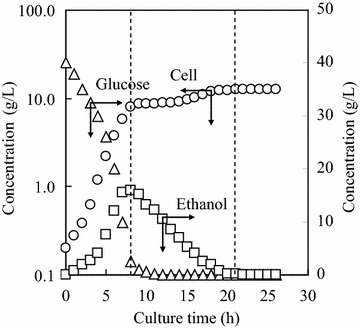
An example of the changes of cell, glucose and ethanol concentrations in culture broth during the aerobic batch culture of yeast AFY (initial glucose concentration: 40.0 g/L)

Figure 1 shows an example of the changes of cell, glucose and ethanol concentrations in culture broth during the aerobic batch culture of yeast AFY, when the culture medium had an initial glucose concentration of 40 g/L. Similar with the other *S. cerevisiae* strains (Serio et al. 2001; Wills 1990; Jones and Kompala 1999), the growth of yeast AFY exhibited a diauxic pattern, with the first growth stage consuming glucose and the second growth stage consuming ethanol. In the first growth stage, glucose was also converted to ethanol via a fermentative metabolic pathway. However, the yeast preferentially consumed glucose when both glucose and ethanol were available, until all the glucose was consumed completely. The change of the first growth stage to the second growth stage related to a switch-over in enzymatic reactions, and the synthesizing of new enzymes (Woehrer and Roehr 1981). When glucose concentration was below a critical level, the yeast might utilize simultaneously glucose and ethanol, based on the respiratory bottleneck (Dantigny 1995).

**Fig. 2:**
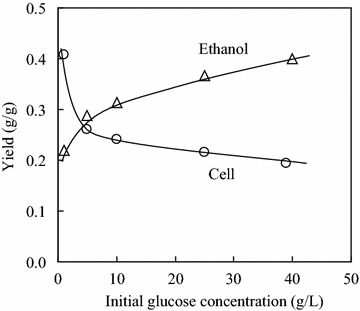
Changes of cell and ethanol yields with initial glucose concentration in the first growth stage of yeast AFY

Figure 2 shows the changes of cell and ethanol yields with initial glucose concentration in the first growth stage of yeast AFY. In Fig. 2, the cell yield (*Y*_C1_) and the ethanol yield (*Y*_E1_) were defined as the cell growth and the ethanol formation per gram of glucose consumption, respectively. *Y*_C1_ decreased with increasing initial glucose concentration. Especially, the decrease was fast in the initial glucose concentration range of 1.0–5.0 g/L. On the other hand, *Y*_E1_ changed in the opposite direction as *Y*_C1_ did. At the initial glucose concentration of 1.0 g/L, *Y*_C1_ was 0.41 g/g, and *Y*_E1_ was 0.22 g/g. When the initial glucose concentration increased to 40.0 g/L, *Y*_C1_ decreased to 0.20 g/g, while *Y*_E1_ increased to 0.40 g/g. The suppression of cell growth (i.e., the Crabtree effect) was due to the repression of respiratory enzyme synthesis, the inactivation of respiratory enzymes and the reduction of glucose transport activity (Win et al. 1996; Serio et al. 2001).

**Fig. 3:**
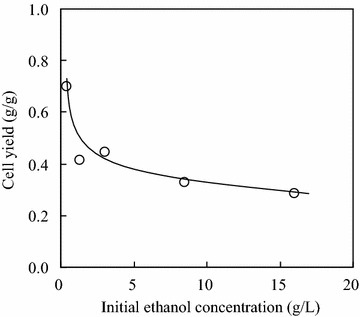
Change of cell yield with initial ethanol concentration in the second growth stage of yeast AFY

Figure 3 shows the change of cell yield (*Y*_C2_) with initial ethanol concentration in the second growth stage of yeast AFY. *Y*_C2_ was defined as the cell growth per gram of ethanol consumption. *Y*_C2_ decreased gradually with increasing initial ethanol concentration. At the initial ethanol concentration of 0.4 g/L, *Y*_C2_ was 0.70 g/g. When the initial ethanol concentration increased to 16.0 g/L, *Y*_C2_ decreased to 0.28 g/g. The decrease of *Y*_C2_ was fast in the initial ethanol concentration range of 0.4–1.3 g/L. The decrease of *Y*_C2_ was caused by the toxicity of ethanol, including the restrictions of metabolism pathways in cells, the reduction of adenosine triphosphatase (ATPase) activity in cell membrane, the change of phospholipid/ergosterol ratio in cell membrane, and the oxidative damage of mitochondria (Zhang et al. 2015).

### Kinetics of cell growth on glucose substrate

In the first growth stage of yeast AFY, the culture media had a constant proportion of nutrients, and constant levels of DO and pH. Therefore, glucose could be considered as the dominating substrate when it was present in the culture media (Serio et al. 2001; Jones and Kompala 1999). Glucose was used to produce cell material and metabolic products, and to maintain cells’ life.

#### Monod equation for cell growth

**Fig. 4:**
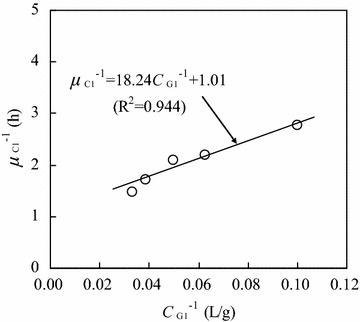
Relationship between the reciprocal of specific cell growth rate and the reciprocal of glucose concentration (initial glucose concentration: 40.0 g/L; glucose concentration range: 2.5–30.0 g/L)

Figure 4 shows the relationship between the reciprocal of specific cell growth rate (*μ*_C1_^−1^) and the reciprocal of glucose concentration (*C*_G1_^−1^) when the initial glucose concentration was 40.0 g/L. In the glucose concentration (*C*_G1_) range of 2.5–30.0 g/L, *μ*_C1_^−1^ increased linearly with increasing *C*_G1_, and their relationship could be expressed by the following Monod equation (a transformation) (Birol et al. 1998; Manikandan et al. 2008):

2 $$\mu_{C1}^{ - 1} = \frac{{K_{1} }}{{\mu_{C1,\hbox{max} } }}C_{G1}^{ - 1} + \frac{1}{{\mu_{C1,\hbox{max} } }} = 18.24C_{G1}^{ - 1} + 1.01\quad(R^{2} = 0.944)$$

Using Eq. 2, it was calculated that *μ*_C1,max_ = 0.99 h^−1^, and *K*_1_ = 10.80 g/L. Furthermore, the cell growth rate on glucose substrate could be described as:

3 $$\frac{{dC_{C1} }}{dt} = \mu_{C1} C_{C1} = \frac{{0.99 \times C_{G1} }}{{10.80 + C_{G1} }} \times C_{C1}$$

It should be noted that Eqs. 2 and 3 were effective in the glucose concentration range of 2.5–30.0 g/L, where the cell growth followed the exponential phase.

#### Luedeking–Piret equation for ethanol formation

**Fig. 5:**
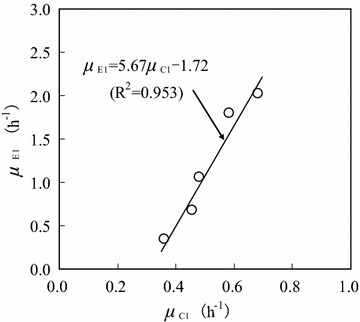
Relationship between specific ethanol formation rate and specific cell growth rate (initial glucose concentration: 40.0 g/L; glucose concentration range: 2.5–30.0 g/L)

Figure 5 shows the relationship between specific ethanol formation rate (*μ*_E1_) and specific cell growth rate (*μ*_C1_) when the initial glucose concentration was 40.0 g/L. The values of *μ*_E1_ and *μ*_C1_ were obtained in the *C*_G1_ range of 2.5–30.0 g/L (the same as that in Fig. 4). *μ*_E1_ increased linearly with increasing *μ*_C1_, following the Luedeking–Piret equation (Manikandan et al. 2008; Shen et al. 2008; Dai et al. 2011):

4 $$\mu_{E1} = \alpha_{E1} \mu_{C1} + \beta_{E1} = 5.67\mu_{C1} - 1.72\quad(R^{2} = 0.953)$$

Using Eq. 4, it was calculated that *α*_E1_ = 5.67, and *β*_E1_ = −1.72. Furthermore, the ethanol formation rate on glucose substrate could be described as:

5 $$\frac{{dC_{E1} }}{dt} = \mu_{E1} C_{C1} = (5.67\mu_{C1} - 1.72)C_{C1}$$

#### Pseudo-Luedeking–Piret equation for glucose consumption

**Fig. 6:**
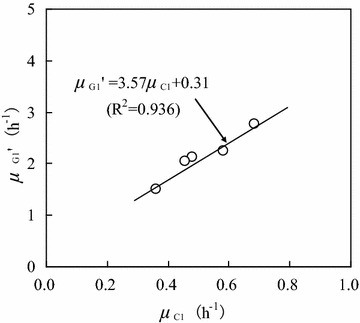
Relationship between specific glucose consumption rate and specific cell growth rate (initial glucose concentration: 40.0 g/L; glucose concentration range: 2.5–30.0 g/L)

Figure 6 shows the relationship between specific glucose consumption rate ($\mu_{G1}^{\prime}$) and specific cell growth rate (*μ*_C1_) when the initial glucose concentration was 40.0 g/L. The values of $\mu_{G1}^{\prime}$ and *μ*_C1_ were obtained in the *C*_G1_ range of 2.5–30.0 g/L (the same as that in Fig. 4). $\mu_{G1}^{\prime}$ increased linearly with increasing *μ*_C1_, following the pseudo-Luedeking–Piret equation (Manikandan et al. 2008; Shen et al. 2008; Dai et al. 2011):

6 $$\mu_{G1}^{\prime} = \omega_{G1} \mu_{C1} + \eta_{G1} = 3.57\mu_{C1} + 0.31\quad(R^{2} = 0.936)$$

Using Eq. 6, it was calculated that *ω*_G1_ = 3.57, and *η*_G1_ = 0.31. Furthermore, the glucose consumption rate could be described as:

7 $$\frac{{dC_{G1} }}{dt} = - \mu_{G1} 'C_{C1} = - (3.57\mu_{C1} + 0.31)C_{C1}$$

#### Comparison of calculated values and experimental data on glucose substrate

**Fig. 7:**
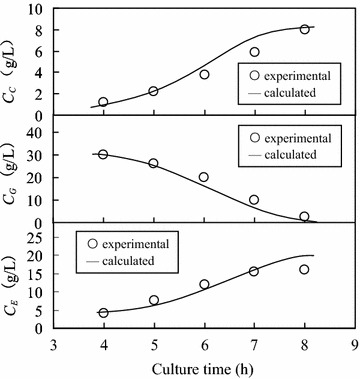
Comparison of calculated values and experimental data for the cell, ethanol and glucose concentrations in culture broth (initial glucose concentration: 40.0 g/L; glucose concentration range: 2.5–30.0 g/L)

In the present study, the simultaneous equations of Eq. 3 (Monod equation), Eq. 5 (Luedeking–Piret equation) and Eq. 7 (pseudo-Luedeking–Piret equation) were solved numerically using the forward difference method (Shinozaki and Matsushita 1976). The time interval in the forward difference analysis was set at 15 min. In Fig. 7, the calculated values are compared with the experimental data for cell, ethanol and glucose concentrations in the culture broth. The aerobic batch culture was started with the initial glucose concentration of 40.0 g/L, and the glucose concentration varied in the range of 2.5–30.0 g/L. The calculated values agreed with the experimental data, indicating that the simultaneous equations established above described well the first exponential growth phase of yeast AFY.

The kinetic analysis was also performed for the aerobic batch culture of yeast AFY with other initial glucose concentrations. It was found that the simultaneous equations of Monod equation, Luedeking–Piret equation and pseudo-Luedeking–Piret equation were applicable to the first exponential growth phase when the initial glucose concentration was in the range of 5.0–40.0 g/L. The values of kinetic parameters and their corresponding glucose concentration ranges for different initial glucose concentrations are listed in Table 2. In each corresponding glucose concentration range, the cells growth followed the exponential phase. For the initial glucose concentration of 5.0, 10.0 and 40.0 g/L, *μ*_C1,max_ was 0.52, 0.98 and 0.99 h^−1^, while *K*_1_ was 0.58, 2.97 and 10.80 g/L, respectively. *μ*_C1,max_ had no correlation with the initial glucose concentration. The kinetic features were also reported previously for the stirred batch fermentation of immobilized yeast *S. cerevisiae* ATCC 9763 (Birol et al. 1998).

**Table 2:** Values of kinetic parameters and their corresponding glucose concentration ranges for different initial glucose concentrations in the first stage

*C*_G0_ (g/L)	*C*_G1_ range (g/L)	*μ*_C1,max_ (h^−1^)	*K*_1_ (g/L)	*α*_E1_ (–)	*β*_E1_ (–)	*ω*_G1_ (–)	*η*_G1_ (–)
5.0	0.1–2.6	0.52	0.58	2.44	−0.44	5.78	−0.58
10.0	0.2–5.0	0.98	2.97	1.06	−0.02	3.70	−0.11
40.0	2.5–30.0	0.99	10.80	5.67	−1.72	3.57	0.31

### Kinetics of the cell growth on ethanol substrate

In the second growth stage of yeast AFY, the cells continued to grow on ethanol substrate after the glucose was depleted (Fig. 1). Assuming that the culture media had enough nutrients besides the ethanol, the ethanol became the dominating substrate, and the culture process on ethanol substrate could be analyzed kinetically in the same way as that on glucose substrate.

#### Monod equation for cell growth

**Fig. 8:**
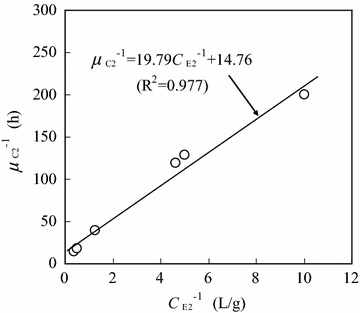
Relationship between the reciprocal of specific cell growth rate and the reciprocal of ethanol concentration (initial ethanol concentration: 16.0 g/L; ethanol concentration range: 0.1–2.6 g/L)

Figure 8 shows the relationship between the reciprocal of specific cell growth rate (*μ*_C2_^−1^) and the reciprocal of ethanol concentration (*C*_E2_^−1^) when the initial ethanol concentration was 16.00 g/L. In the ethanol concentration (*C*_E2_) range of 0.1–2.6 g/L, *μ*_C2_^−1^ increased linearly with increasing *C*_E2_^−1^, and their relationship could be expressed by the following Monod equation (a transformation) (Birol et al. 1998; Manikandan et al. 2008):

8 $$\mu_{C2}^{ - 1} = \frac{{K_{2} }}{{\mu_{C2,\hbox{max} } }}C_{E2}^{ - 1} + \frac{1}{{\mu_{C2,\hbox{max} } }} = 19.79C_{E2}^{ - 1} + 14.76 \quad(R^{2} = 0.977)$$

Using Eq. 8, it was calculated that *μ*_C2,max_ = 0.07 h^−1^, and *K*_2_ = 1.34 g/L. Furthermore, the cell growth rate could be described as:

9 $$\frac{{dC_{C2} }}{dt} = \mu_{C2} C_{C2} = \frac{{0.07 \times C_{E2} }}{{1.34 + C_{E2} }}C_{C2}$$

It should be noted that Eq. 9 was effective in the ethanol concentration range of 0.1–2.6 g/L, where the cell growth followed the exponential phase.

#### Pseudo-Luedeking–Piret equation for ethanol consumption

**Fig. 9:**
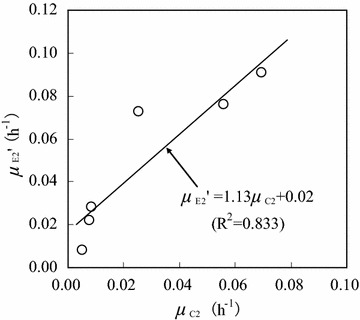
Relationship between specific ethanol consumption rate and specific cell growth rate (initial ethanol concentration: 16.0 g/L; ethanol concentration range: 0.1–2.6 g/L)

Figure 9 shows the relationship between specific ethanol consumption rate ($\mu_{E2}^{\prime}$) and specific cell growth rate (*μ*_C2_) when the initial ethanol concentration was 16.0 g/L. The values of $\mu_{E2}^{\prime}$ and *μ*_C2_ were obtained in the *C*_E2_ range of 0.1–2.6 g/L (the same as that in Fig. 8). $\mu_{E2}^{\prime}$ increased linearly with increasing *μ*_C2_, following the pseudo-Luedeking–Piret equation (Manikandan et al. 2008; Shen et al. 2008; Dai et al. 2011):

10 $$\mu_{E2}^{\prime} = \omega_{E2} \mu_{C2} + \eta_{E2} = 1.13\mu_{C2} + 0.02\quad(R^{2} = 0.833)$$

Using Eq. 10, it was calculated that *ω*_E2_ = 1.13, and *η*_E2_ = 0.02. Furthermore, the ethanol consumption rate could be described as:

11 $$\frac{{dC_{E2} }}{dt} = - \mu_{E2}^{\prime} C_{C2} = - (1.13\mu_{C2} + 0.02)C_{C2}$$

#### Comparison of calculated values and experimental data on ethanol substrate

**Fig. 10:**
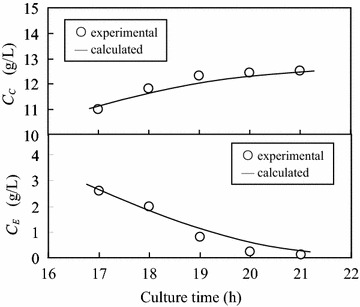
Comparison of calculated values and experimental data for the cell and ethanol concentrations in culture broth (initial ethanol concentration: 16.0 g/L; ethanol concentration range: 0.1–2.6 g/L)

The simultaneous equations of Eq. 9 (Monod equation) and Eq. 11 (pseudo-Luedeking–Piret equation) were solved numerically using the forward difference method (Shinozaki and Matsushita 1976). The time interval in the forward difference analysis was also set at 15 min as that in section “Comparison of calculated values and experimental data on ethanol substrate”. In Fig. 10, the calculated values are compared with the experimental data for cell and ethanol concentrations in the culture broth. The aerobic batch culture was started with the initial ethanol concentration of 16.0 g/L, and the ethanol concentration varied in the range of 0.1–2.6 g/L. The calculated values agreed with the experimental data, indicating that the simultaneous equations established above described well the second exponential growth phase of yeast AFY.

The simultaneous equations of Monod equation and pseudo-Luedeking–Piret equation could also be used to describe the second exponential growth phase with other initial ethanol concentrations. The values of kinetic parameters and their corresponding ethanol concentration ranges for different initial ethanol concentrations are listed in Table 3. The initial ethanol concentration of 1.3, 3.0 and 16.0 g/L was resulted from the aerobic batch culture with the initial glucose concentration of 5.0, 10.0 and 40.0 g/L (as shown in Table 2), respectively. For the initial ethanol concentration of 1.3, 3.0 and 16.0 g/L, *μ*_C2,max_ was 0.11, 0.06 and 0.07 h^−1^, while *K*_2_ was 0.47, 0.67 and 1.34 g/L, respectively. In comparison with *μ*_C1,max_ (0.52, 0.98, 0.99 h^−1^), *μ*_C2,max_ (0.11, 0.06, 0.07 h^−1^) was much lower. The reduction of specific cell growth rate in the second growth phase was due to the metabolic difference of glucose and ethanol (Wills 1990), and the changes of nutrient and cell concentrations in the culture broth, etc. These results indicated that the efficiency of yeast production could be improved by reducing ethanol production in the first growth stage. For this purpose, fed-batch culture with a glucose-limited feed rate is usually applied.

**Table 3:** Values of kinetic parameters and their corresponding ethanol concentration ranges for different initial ethanol concentrations in the second stage

*C*_E0_ (g/L)	*C*_E2_ range (g/L)	*μ*_C2,max_ (h^−1^)	*K*_2_ (g/L)	*ω*_E2_ (–)	*η*_E2_ (–)
1.3	0.1–0.5	0.11	0.47	0.06	0.07
3.0	0.7–2.0	0.06	0.67	1.96	0.03
16.0	0.1–2.6	0.07	1.34	1.13	0.02

It should be noted that the values of maximum specific growth rate (μ_C,max_) and substrate saturation constant (K) vary with the metabolism of yeast cells, which are affected by yeast strain, growth history, culture condition, etc. The two parameters are interrelated. When the value of a parameter changes, the value of another parameter will also change (Birol et al. 1998; Wang 2004; Kostov et al. 2012). In addition, different values might be obtained for *μ*_C,max_ and K when different kinetic models are used (Kostov et al. 2012). Therefore, the parameter values obtained for the baker’s yeast *S. cerevisiae* AFY in the present study are valid only for the processes under similar conditions.

## Conclusions

Aerobic batch culture of the baker’s yeast *S. cerevisiae* AFY was carried out using culture media with various initial glucose concentrations, and the culture process was discussed kinetically. The experimental results could be concluded as follows:

1. The growth of yeast AFY exhibited a diauxic pattern, with the first growth stage consuming glucose and the second growth stage consuming ethanol. The cell yield decreased with increasing initial glucose concentration in the first growth stage, and also decreased with increasing initial ethanol concentration in the second growth stage. The efficiency of yeast production could be improved by reducing ethanol production in the first growth stage.
2. In the initial glucose concentration range of 5.0–40.0 g/L, the simultaneous equations of Monod equation, Luedeking–Piret equation and pseudo-Luedeking–Piret equation described well cell, ethanol and glucose concentrations in either of the two exponential growth phases.
3. At the initial glucose concentration of 5.0, 10.0 and 40.0 g/L, the first exponential growth phase had a maximal specific cell growth rate of 0.52, 0.98 and 0.99 h^−1^, while the second exponential growth phase had a maximal specific cell growth rate of 0.11, 0.06 and 0.07 h^−1^, respectively.

## List of symbols

*A*: optical density of culture broth at 600 nm (–)
*C*_C_: cell concentration of culture broth (g/L)
*C*_C1_: cell concentration of culture broth in the first growth stage (g/L)
*C*_C2_: cell concentration of culture broth in the second growth stage (g/L)
*C*_E0_: initial ethanol concentration of culture broth in the second growth stage (g/L)
*C*_E1_: ethanol concentration of culture broth in the first growth stage (g/L)
*C*_E2_: ethanol concentration of culture broth in the second growth stage (g/L)
*C*_G0_: initial glucose concentration of culture broth in the first growth stage (g/L)
*C*_G1_: glucose concentration of culture broth in the first growth stage (g/L)
*K*_1_: substrate saturation constant for glucose in the first growth stage (g/L)
*K*_2_: substrate saturation constant for ethanol in the second growth stage (g/L)
*t*: culture time (h)
*Y*_C1_: cell yield in the first growth stage (g/g)
*Y*_C2_: cell yield in the second growth stage (g/g)
*Y*_E1_: ethanol yield in the first growth stage (g/g)
*α*_E1_: Luedeking–Piret equation parameter for growth-associated ethanol formation in the first growth stage (–)
*β*_E1_: Luedeking–Piret equation parameter for non-growth-associated ethanol formation in the first growth stage (–)
*η*_E2_: pseudo-Luedeking–Piret equation parameter for non-growth-associated ethanol consumption in the second growth stage (−)
*η*_G1_: pseudo-Luedeking–Piret equation parameter for non-growth-associated glucose consumption in the first growth stage (−)
*μ*_C1_: specific cell growth rate in the first growth stage (h^−1^)
*μ*_C1,max_: maximal specific cell growth rate in the first growth stage (h^−1^)
*μ*_C2_: specific cell growth rate in the second growth stage (h^−1^)
*μ*_C2,max_: maximal specific cell growth rate in the second growth stage (h^−1^)
*μ*_E1_: specific ethanol formation rate in the first growth stage (h^−1^)
$\mu_{E2}^{\prime}$: specific ethanol consumption rate in the second growth stage (h^−1^)
$\mu_{G1}^{\prime}$: specific glucose consumption rate in the first growth stage (h^−1^)
*ω*_E2_: pseudo-Luedeking–Piret equation parameter for growth-associated ethanol consumption in the second growth stage (–)
*ω*_G1_: pseudo-Luedeking–Piret equation parameter for growth-associated glucose consumption in the first growth stage (–)

## References

[CR1] Aguilar-Uscanga MG, Garcia-Alvarado Y, Gomez-Rodriguez J, Phister T, Delia ML, Strehaiano P (2011). Modelling the growth and ethanol production of *Brettanomyces bruxellensis* at different glucose concentrations. Lett Appl Microbiol.

[CR2] Barrera-Martínez I, González-García RA, Salgado-Manjarrez E, Aranda-Barradas JS (2011). A simple metabolic flux balance analysis of biomass and bioethanol production in *Saccharomyces cerevisiae* fed-batch cultures. Biotechnol Bioprocess Eng.

[CR3] Bertilsson M, Andersson J, Lidén G (2008). Modeling simultaneous glucose and xylose uptake in *Saccharomyces cerevisiae* from kinetics and gene expression of sugar transporters. Bioprocess Biosyst Eng.

[CR4] Birol G, Doruker P, Kardar B, Onsan ZI, Ülgen K (1998). Mathematical description of ethanol fermentation by immobilised *Saccharomyces cerevisiae*. Process Biochem.

[CR5] Chen J (2015) Studies of the growth and fermentation characteristics of frozen-tolerant yeast AFY-1. MD. thesis, Nanjing Tech University, Nanjing, China (**in Chinese**)

[CR6] Chen J, Miao Y, You Y, Ji M, Xu L (2016) Stain homology and fermentability of the freeze-tolerant yeast AFY-1 (in Chinese). J Chin Inst Food Sci Technol (in press)

[CR7] Dai Z, Yin Y, Ruan Z (2011). Microbial fermentation kinetic model and its paramenters estimation by software. Comput Appl Chem.

[CR8] Dantigny P (1995). Modeling of the aerobic growth of *Saccharomyces cerevisiae* on mixtures of glucose and ethanol in continuous culture. J Biotechnol.

[CR9] Jones KD, Kompala DS (1999). Cybernetic model of the growth dynamics of *Saccharomyces cerevisiae* in batch and continuous cultures. J Biotechnol.

[CR10] Kostov G, Popova S, Gochev V, Koprinkova-Hristovab P, Angelovd M, Georgievae A (2012). Modeling of batch alcohol fermentation with free and immobilized yeasts *Saccharomyces cerevisiae* 46 EVD. Biotechnol Biotechnol Equip.

[CR11] Li Y, Zhou C, Ding L, Sun Y, Yang J (2011). Strategies for optimization of fermentation medium composition performance. J Beijing Union Univ.

[CR12] Lisha KP, Sarkar D (2014). Dynamic flux balance analysis of batch fermentation: effect of genetic manipulations on ethanol production. Bioprocess Biosyst Eng.

[CR13] Manikandan K, Saravanan V, Viruthagiri T (2008). Kinetics studies on ethanol production from banana peel waste using mutant strain of *Saccharomyces cerevisiae*. Indian J Biotechnol.

[CR14] Miao Y, Shi X, Chen L, Chen J (2014) A baker’s yeast strain *Saccharomyces cerevisiae* with high freeze tolerance and its application in frozen-dough processing. China: ZL 2013 1 0135021. X

[CR15] Sainz J, Pizarro F, Pérez-Correa JR, Agosin E (2003). Modeling of yeast metabolism and process dynamics in batch fermentation. Biotechnol Bioeng.

[CR16] Serio MD, Tesser R, Santacesaria E (2001). A kinetic and mass transfer model to simulate the growth of baker’s yeast in industrial bioreactors. Chem Eng J.

[CR17] Serio MD, Alteriis ED, Parascandola P, Santacesaria E (2001). A general kinetic and mass transfer model to simulate the baker’s yeast growth in bioreactors. Catal Today.

[CR18] Shen J, Li Y, Shen G, Yang D (2008). Study and application of the kinetics of *Saccharomyces cerevisiae* HYS98 batch fermentation for high productivity of SAM (in Chinese). Chem Bioeng.

[CR19] Shi X, Miao Y, Chen JY, Chen J, Li W, He X, Wang J (2014). The Relationship between freeze tolerance and intracellular compounds in baker’s yeasts. Appl Biochem Biotechnol.

[CR20] Shinozaki T, Matsushita Y (1976). Elementary applied numerical computation for engineering.

[CR21] Wang L (2004). Organic chemistry of pollution.

[CR22] Wang Y, He N, Li Q, Deng X, Lu Y (2007). Optimization of high cell density cultivation conditions of *Saccharomyces cerevisiae*. Ind Microbiol.

[CR23] Wang F, Wu H, Yu Y, Li Q, Zhu J, Dong S, Tang Y (2010). High density fermentation of *Saccharomyces cerevisiae* YQ-7. China Brew.

[CR24] Wills C (1990). Regulation of sugar and ethanol metabolism in *Saccharomyces cerevisiae*. Biochem Mol Biol.

[CR25] Win SS, Impoolsup A, Noomhorm A (1996). Growth kinetics of *Saccharomyces cerevisiae* in batch and fed-batch cultivation using sugarcane molasses and glucose syrup from cassava starch. J Ind Microbiol.

[CR26] Woehrer W, Roehr M (1981). Regulatory aspects of bakers’ yeast metabolism in aerobic fed-batch cultures. Biotechnol Bioeng.

[CR27] Zhang Q, Wu D, Lin Y, Wang X, Kong H, Tanaka S (2015). Substrate and product inhibition on yeast performance in ethanol fermentation. Energy Fuels.

